# Bone density in patients with late onset Pompe disease

**DOI:** 10.5812/ijem.4967

**Published:** 2012-09-30

**Authors:** George Papadimas, Gerassimos Terzis, Constantinos Papadopoulos, Anna Areovimata, Konstantinos Spengos, Stavros Kavouras, Panagiota Manta

**Affiliations:** 1 Department of Neurology, University of Athens, School of Medicine, Eginition Hospital, Athens, Greece; 2 Athletics Laboratory, School of Physical Education and Sport Science, University of Athens, Athens, Greece; 3 Laboratory of Nutrition and Clinical Dietetics, Harokopio University of Athens, Athens, Greece

**Keywords:** Bone Density, α-glucosidase, Pompe Disease

## Abstract

**Background:**

Pompe disease is an inherited metabolic disorder characterized by α-glycosidase deficiency, which leads to lysosomal glycogen accumulation in many different tissues. The infantile form is the most severe with a rapidly fatal outcome, while the late onset form has a greater phenotypic variability, characterized by skeletal muscle dysfunction and early respiratory involvement. Bone mineral density (BMD) has been recently reported to be reduced in many patients with both forms of the disease. Enzyme replacement therapy (ERT) is now available with an undefined, impact on BMD in patients with late onset disease.

**Objectives:**

The present study aimed to investigate BMD in patients with late onset form of Pompe disease before and after ERT initiation.

**Patients and Methods:**

Dual x-ray absorptiometry (DEXA) was examined in four newly diagnosed patients with late onset Pompe disease and in four adults under ERT before and after ERT initiation with a treatment duration of 18 to 36 months.

**Results:**

The initial DEXA showed normal total body BMD z-score in all the patients, while L2-L4 and femoral neck BMD was reduced in three and two patients, respectively. After ERT administration, two patients had an improvement in L2-L4 lumbar spine and one patient in femoral neck BMD z-score with values within normal range.

**Conclusions:**

The results suggested that regional BMD may moderately reduce in some patients with the late onset form of Pompe disease, although profound osteopenia was not observed. The improvement of measurements in L2-L4 and femoral neck BMD z-score in some patients with low pre-treatment values after ERT administration needs to be confirmed in larger scale studies.

## 1. Background

Pompe disease is a recessively inherited lysosomal storage disease caused by total or partial acid α-glucosidase (GAA) deficiency which leads to glycogen accumulation in many tissues. Disease manifestations and the clinical course differ according to onset age , organ involvement extent, and GAA residual activity levels seem to determine the resulting phenotype partially (1, 2). Patients with the infantile form are the most severely affected group, and exhibit the first symptoms soon after birth, while the adult form is milder, presenting with progressive proximal muscle weakness (3, 4) and early respiratory involvement (5). The administration of enzyme replacement therapy (ERT) (Genzyme Corporation, Cambridge, MA, USA) has decisively improved the natural course of the disease in infants and has reversed the fatal outcome (6). Furthermore, a growing body of evidence suggest that ERT may stabilize or even slightly improve the clinical performance of patients with the late onset form of the disease, although concrete conclusions cannot still be reached (3, 7, 8).

Osteoporosis is a common multifactorial skeletal disorder which results from an imbalance in bone remodelling and increases the risk of fractures. The level of exercise is a leading factor for peak bone mass modulation (9). There are recent studies confirming a diminished bone mineral density (BMD) in both children and adult patients which may be attributed to the reduction of mechanical load placed on bones by the weakened muscles (4, 10).

## 2. Objectives

The present study aimed to assess the bone mineral status in newly diagnosed patients with the late onset form of the disease before starting treatment and to investigate whether it may be positively influenced in patients under ERT.

## 3. Patients and Methods

Four newlate onset patients (patients 5 – 8) recently diagnosed with Pompe disease (3 males, 1 female), and four old patients (patients 1 – 4) who have been receiving ERT systematically for up to 34 months were included in the present study (Table 1). Patients who were wheelchair bound or patients under ERT who did not receive regular intravenous infusions, were excluded from the present survey. Muscle strength was measured by manual muscle testing according to the MRC (Medical Research Council) scale (Grade 5: Muscle contracts normally against full resistance, Grade 4: Muscle strength is reduced but muscle contraction can still move joint against resistance, Grade 3: Muscle strength is further reduced such that the joint can be moved only against gravity with the examiner’s resistance completely removed, Grade 2: Muscle can move only if the resistance of gravity is removed, Grade 1: only a trace or flicker of movement is seen, Grade 0: No movement is observed) (11). A sum-score ranging from 0-40 was calculated for each patient by bilateral examination of shoulder abductors, elbow flexors, hip flexors and knee extensors. The stage of the disease was assessed by the modified Walton muscle function scale (Grade 0: Preclinical. All activities normal, Grade 1: Walks normally. Unable to run freely, Grade 2: Detectable defect in posture or gait. Climbs stairs without using the banister, Grade 2.5: Sometimes needs to use bannister to climb stairs, Grade 3: Climbs stairs only with the banister, Grade 3.5: Sometimes climbs stairs with the banister, but at other times unable to climb stairs even with banister, Grade 4: Walks without assistance. Unable to climb stairs, Grade 5: Walks without assistance. Unable to rise from a chair, Grade 6: Walks only with callipers or other aids, Grade 7: Wheelchair bound) (12). The diagnosis was based on the deficient GAA activity in skin cultured fibroblasts, and genomic DNA mutational analysis isolated from peripheral blood leukocytes (Table 1).

**Table 1:** Patients’ Characteristics at the Time of Diagnosis

**Patient**	**Sex**	**Age, y**	**Manual Muscle Testing Score**	**Severity Score a**	**Mutation**	**α-Glucosidase in Fibroblasts nmoles, mg.min^-1^**	**Clinical Characteristics**
1	F	71	30	3	IVS1-13 T > G and 925G > A (GlyC309Arg)	0.10	Easy fatigability since early adulthood and proximal weakness by the age of 60
2	F	46	29	2,5	IVS1-13 T > G (homozygous)	0.21	Proximal weakness since late adolescence
3	M	40	37	2	IVS1-13 T > G and c.2066-2070dup.	0.26	Waddling gait since the last decade
4	F	46	34	2	IVS1-13 T > G and 2431insC (GAA ex17)	0,08	Respiratory insufficiency and proximal weakness since early adulthood
5	M	41	36	2	IVS1-13 T > G and 1293del20 (GAA ex8)	0,16	Waddling gait and respiratory insufficiency since the last decade
6	F	43	38	0	IVS1-13 T > G and 1293del20 (GAA ex8)	0,25	Asymptomatic hyperCkemia – Positive family history
7	M	41	34	2	IVS1-13 T > G and R870X, 2608C > T (GAA ex18)	0,18	Easy fatigability, respiratory insufficiency and proximal weakness since the last decade
8	M	21	40	0	IVS1-13T > G and 1943G > A (GAA ex14)	0,24	Asymptomatic hyperCkemia – Positive family history

^a^Severity score according to modified Walton scale of muscle function

Body composition analysis was performed by dual x-ray absorptiometer (DXA model DPX-L, LUNAR Radiation, Madison, WI, USA) and was analyzed by the LUNAR Radiation body composition program in all the patients under study. Fat mass, LBM and BMD were measured for the total body, the lumbar spine (L2-L4) and femoral neck, respectively. No approval was needed by Ethics Committee, as this test is routinely performed in patients with muscle diseases. Two different investigators performed all analyses and the mean value was used for further analyses. Based on the World Health Organization standards, all subjects with BMD z score greater than -1 were considered as normal, those with BMD z score between -1 and -2,5 were diagnosed as osteopenic and those with BMD z score less than -2,5 were diagnosed as having osteoporosis (13). The measurements were repeated in the four patients under ERT with alpha-glucosidase (Myozyme, Genzyme, USA) that was administered intravenously at a rate of 20 mg.kg^-1^ biweekly (14). No significant side effects were reported during the infusions.

DXA measurements (baseline values from all the patients and the last measurements from the four old patients under ERT) are shown in Table 2.

**Table 2:** Patients’ Body Composition Measurements

Patient	Time of ERT Administratio n, Mo	Total BMD a				L2-L4 BMD a				Femoral Neck BMD a				Total LBM a		Fat	
		**Pre-ERT** a		**cost-ERT** a		**Pre-ERT** a		**Post-ERT** a		**Pre-ERT** a		**Post-ERT** a		**Pre-ERT** a	**Post-ERT** a	**Pre-ERT** a	**Post-ERT **a
		z score	areal g/cm^2^	z score	areal g/cm^2^	z score	areal g/cm^2^	z score	areal g/cm^2^	z score	areal g/cm^2^	z score	areal g/cm^2^	g	g	%	%
1	32	-0,2	1,044	1,0	1,065	-1,6	0,742	1,1	1,045	0,6	0,875	1,1	0,924	33661	32107	53	54,5
2	34	1,1	1,308	0,9	1.264	3,6	1,571	4,4	1,633	-1,2	1,056	-0,9	0,902	38132	37108	55,6	54,9
3	25	-0,6	1,166	-0,5	1,177	1,7	1,446	2,3	1,515	-0,5	1,045	-0,4	0,968	46141	51654	36,2	26,8
4	19	-1,0	0,985	-1,0	0,982	-1,3	0,985	-0,1	1,304	-2,4	0,621	-1,6	0,897	27761	27999	36,7	41,5
5	-	0,3	1,177	-	-	1.6	1,378	-	-	-0.4	1,056	-	-	51115	-	34,1	-
6	-	0.2	1,078	-	-	-1.2	1,007	-	-	0.4	0,958	-	-	30642	-	31.4	-
7	-	-0,1	1,180	-	-	1.1	1,369	-	-	-0.5	0,922	-	-	42073	-	25.9	-
8	-	0,6	1,290	-	-	1.2	1,389	-	-	-0.2	1,043	-	-	45737	-	42.5	-

^a^Abbreviations: ERT, Enzyme replacement therapy; BMD, Bone mineral density; LBM, Lean body mass

### 3.1. Statistical Analysis

Means ± SD were used to describe variables. Dependent student t-test was employed to investigate differences in body composition before and after one year of enzyme replacement therapy in late onset Pompe disease patients. P ≤ 0.05 was used as a two tail level of significance.

## 4. Results

The present study consisted of four newly diagnosed patients (three males and one female) with the late onset form of Pompe disease and four patients (one male and three females) under ERT for at least more than 18 months. All the patients had a baseline DXA scan performed after the confirmation of the diagnosis. The most common mutation c.-32-13T > G (IVS1-13T > G) was present in all of them and their clinical characteristics are summarized in Table 1.

All the patients had normal total-body BMD z-score at baseline. Lumbar spine (L2-L4) and femoral neck BMD were in the range of osteopenia in three (1, 4 and 6) and two patients (2 and 4), respectively. Their BMD values and soft tissue measurements (lean body mass and body fat percentage) are shown in Table 2.

The administration of ERT did not substantially change the total body BMD measurements in the four patients under treatment. However, L2-L4 BMD was normalized in the two patients (1 and 4) who had initially lumbar osteopenia, while femoral neck BMD fell within the range of normal reference values in patient 2, who had previously osteopenic measurements. Total lean body mass substantially increased after 25 months under ERT in patient 3 (pre-ERT: 46141g vs post-ERT: 51654g, Table 2), who showed at the same time a remarkable body fat reduction (pre-ERT: 36.2% vs post-ERT: 26.8%, Table 2). On the contrary, body fat was increased in patient 4 after 19 months of treatment, while lean body mass remained practically unaltered.

## 5. Discussion

Osteoporosis is a progressive systemic skeletal disorder characterized by low BMD, and bone micro architecture deterioration (13). Normal muscle function is required to maintain a healthy skeleton. A plethora of neuromuscular diseases that affect muscle strength, such as myasthenia gravis, Duchenne muscular dystrophy or spinal cord injury result in diminished bone properties (15). There are recent studies supporting the hypothesis that low BMD may be a frequent finding in adult patients with Pompe disease due to lower mechanical load applied on bones by the weakened muscles (4, 10). In the current study, DXA was used to measure BMD and soft tissue composition in patients with the late onset form of Pompe disease. Although this technique has some limitations, such as the inability to measure cortical area and a real volumetric bone density (17), it remains a common and reliable method to evaluate bone mineral status and is used to diagnose osteoporosis and to predict fracture risk (18). The results reconfirmed that osteopenia may be present at diagnosis time in bones which are adjacent to the insertion of affected muscles, such as femur and lumbar spine. Muscle weakness reduces the application of mechanical forces on the skeleton, which are essential for bone remodelling and adaptation to keep high bone strength (17). Similarly, BMD in the proximal femur but not the lumbar spine is severely diminished in boys with Duchenne muscular dystrophy early in the disease course before ambulation is significantly affected (19).

Despite the fact that low BMD values at femoral neck and L2-L4 lumbar spine may be causally associated with the strength diminution in the proximal part of lower extremities and paraspinal muscles, no statistical correlation seems to exist (data not shown). A reasonable explanation could be that other factors can influence bone health. BMD is primarily influenced by everyday activity level of various body parts (20), hormonal profile (21),and gender and age (22). The present study patients followed a sedentary lifestyle with a rather low level of physical activity at the time of the initial body composition measurement. The age might not affect BMD except in patient 1 who was 71 years old at baseline measurements and she had reduced L2-L4 BMD z score values within the range of osteopenia.

There is an increasing body of scientific data suggesting that ERT may improve muscle strength and respiratory function in some patients with the late onset form of the disease (3, 7, 8, 21). Thus, it was investigated whether ERT administration could have a positive effect on BMD in patients who received treatment biweekly for 19 to 34 months (Table 2). The slight tendency towards L2-L4 improvement and femoral neck BMD z-score in some patients with low pretreatment BMD values compatible with regional osteopenia can not substantiate per se a possible ERT effect on bone metabolism, and further studies in a larger scale of patients are needed before reaching final conclusions. Moreover, the absence of remarkable changes in BMD before and after treatment may be reasonably attributed to lack of alterations in physical activity levels of patients during ERT.

Based on the results of the current study, patients with Pompe disease seem to be at greater risk for osteoporosis (4, 10). Although osteopenia does not result in fractures by itself, it may increase traumatic fractures especially when combined with physical impairment and a higher incidence of falls as in patients with an underlying myopathy (25). Dual x-ray absorptiometry is a common laboratory method to assess body composition (23), which may be valuable either to monitor changes associated with the disease or to evaluate the effectiveness of alternative therapeutic interventions such as nutrition program and exercise training.

In line with previous studies, the current study suggests that regional BMD in femoral neck and lumbar spine may be moderately reduced in patients with late onset form of Pompe disease, although it is important to emphasize that the small sample size of the study prevented the use statistical tests and limited the ability to generalize the findings. It is recommended that longitudinal studies are undertaken to ascertain the extent that the low mechanical stress applied on bones by the weak muscles is responsible for the diminished mineral density and whether alterations of extrinsic materials properties, such as bone geometry and bone mass may also co-exist.
